# Onset of lupus like syndrome in patients with spondyloarthritis treated with anti-TNF-α

**DOI:** 10.1186/1755-7682-5-7

**Published:** 2012-02-15

**Authors:** Eduardo Puertas-Abreu, Elaudi Rodríguez Polanco, Miriam Azocar, Luis Antonio Mundarain, Concepción Morelia Nuñez-Sotelo, Rafael Montaño, Freddy Herrera Vivas, Zair Tovar Noguera, Francisco Marín, Omar Bellorin, José Gregorio Rivas, Eduardo Toro, Eugenia Benzaquen, Mayra Rauseo, Luis Arturo Gutiérrez González

**Affiliations:** 1 Hospital Central de Cumaná "Dr. Antonio Patricio de Alcalá", Final Av. Bolívar, Ciudad Cumana 6101, Estado Sucre, Venezuela; 2 Hospital IVSS "Dr. Rafael Calles-Sierra", Av. Táchira con Av. Ínter comunal Lagoven, Ciudad Punto Fijo Código Postal 4102, Edo. Falcón, Estado Falcón, Venezuela; 3 Hospital Central de Maturín "Dr. Manuel Núñez y Tovar", Avenida Bicentenario, Ciudad Maturín 6201, Estado Monagas. País, Venezuela; 4 Hospital Central de Ciudad Bolívar "Dr. Ruiz y Páez", 8001 Estado Bolívar, País, Venezuela; 5 Hospital Central de Barquisimeto "Dr. Antonio María Pineda", Av. Dr. Jose María Vargas, Ciudad Barquisimeto 3001, Edo. Lara, Venezuela; 6 Hospital Central de Carupano "Dr. Santos Anibal Dominicci", Avenida Circunvalación oeste, Ciudad Carúpano 6150, Edo. Sucre, Venezuela; 7 Hospital Universitario de Caracas. HUC-UCV, Urb. Los Chaguaramos, Ciudad Universitaria, Caracas 1020, DC, Venezuela; 8 Hospital de la Isla de Margarita "Dr. Luis Ortega", Av. 4 de Mayo, Ciudad Porlamar, Nueva Esparta 6301, Venezuela; 9 Centro Nacional de Enfermedades Reumáticas (CNER), Hospital Universitario de Caracas, Urbanización Los Chaguaramos, Ciudad universitaria UCV, Caracas 1020ª, Venezuela

**Keywords:** Antinuclear antibodies, Lupus-like syndrome, Anti-TNFα therapy, Anti-dsDNA, Psoriasis

## Abstract

**Background:**

The anti-TNFα therapy has been since its approval by the FDA, along with nonsteroidal antiinflammatory drugs (NSAIDs), one of the most important therapies for control of spondyloarthritis (SpA). The onset of Lupus Like Syndrome (LLS) has been described in patients with rheumatoid arthritis (RA) treated with anti-TNFα therapy but there is little literature on the occurrence of this entity in patients with SpA.

**Methods:**

We studied 57 patients with SpA who received more than 1 year of anti-TNFα therapy (infliximab, adalimumab or etanercept). Patients were analyzed for the development of LLS, in addition to measuring ANA levels ≥ 1:160 and Anti-dsDNA (measured by IIF).

**Results:**

In total, 7.01% of patients treated with anti-TNFα had titers of ANA ≥ 1:160, whereas 3.5% of patients had serum levels of dsDNA. However, only one patient (1.75%; n = 1) experienced clinical symptoms of LLS; this was a female patient with a history of psoriatic arthritis.

**Conclusions:**

The presence of LLS secondary to anti-TNFα therapy in patients with SpA is observed less frequently compared with patients with RA. LLS was only detected in a patient with a history of psoriasis since youth, who developed psoriatic arthritis after 27 years of age and had received anti-TNFα therapy for > 2 years. This may be because LLS is an entity clearly associated with innate immunity, with little central role of B and T cells.

## Introduction

Lupus Like Syndrome (LLS) is an autoimmune disease characterized by the appearance of at least one serological marker and one non serological marker of systemic lupus erythematosus (SLE) (according to ACR criteria) in patients who previously had not; the syndrome commonly is associated with use of drugs such as procainamide and hydralazine, [1-3] although other causes exist, for example, in the context of a paraneoplastic syndrome (malignant neoplasms) [4-6]. After approval by the FDA of anti-TNFα therapy in RA and rheumatic diseases to such as spondyloarthritis, the emergence of this syndrome has been described under the name of lupus-like syndrome [7-10] and other ones under the name DILE (drug-induced lupus erythematosus), but classification criteria are not yet well defined and, worse still, there are few published data on its occurrence in spondyloarthritis [8,9].

**Table 1:** Clinical and demographic data of the cohort (n = 57)

Characteristics	N = 57 (% or range)
Male/Female (%)	39/18 (68.4/31.6)

Mean Age (range)	41.2 (23-66)

Type of Spondyloarthritis (%)	

Ankylosing Spondylitis	8 (14)

Reactive arthritis	16 (28)

Undiffenciated Spondyloarthritis	22 (38.6)

Psoriatic arthritis	7 (12.3)

Enteropathic artritis	4 (7.1)

Patients treated with IFX (%)	28 (49.1)

Patients treated with ADA (%)	19 (33.3)

Patients treated with ETP (%)	10 (17.6)

Mean duration of therapy with IFX in months (range)	18 (1-78)

Mean duration of therapy with ADA in months (range)	13 (1-88)

Mean duration of therapy with ETP in months (range)	7 (1-38)

ANA positive (%)	4 (7.01)

Anti dsDNA (%)	2 (3.5)

Moderate lupus-like symptoms (%)	0

Severe lupus-like symptoms (%)	1

The mechanism by which the therapy against tumor necrosis factor anti-TNFα induces LLS is not yet well understood [10]. One hypothesis states that the binding of anti-TNF drugs to the cell surface containing TNF induces apoptosis of cells, causing the release of nucleosomal autoantigens and induction of anti-dsDNA [11-16]. A second hypothesis is that suppression of T-h1 cells by anti-TNF therapy will generate an exuberant T-h2 response, leading to overproduction of auto-antibodies [5,17]. A third hypothesis suggests that using an immunosuppressant as anti-TNF associated with disease-modifying drugs (DMARD), patients are more prone to bacterial infections, stimulating polyclonal B cell activation and therefore the production of auto-antibodies [18-21].

The anti-TNFα therapy is one of the most important therapies, along with nonsteroidal antiinflammatory drugs (NSAIDs), for control of spondyloarthritis (SpA), but still is not classified as disease-modifying drugs (since it does not alter the disease), but it is classified as DC-ART (disease controlling antirheumatic therapy). The onset of LLS in patients with rheumatoid arthritis (RA) using anti-TNF therapy has been described, but there is little literature on the occurrence of this entity in patients with SpA [22-25].

## Patients and methods

This is a multicenter, retrospective study that enrolled 57 patients from our rheumatology specialized clinic. The ethics committee of hospital Universitario de Cararas approved the study in accordance with the Declaration of Helsinki.

Where all medical histories of patients with spondyloarthritis were reviewed according to the ASAS classification and the modified New York criteria; our patients had received more than 1 year of anti-TNFα biologic therapy: infliximab, adalimumab and etanercept. We recorded the duration of the disease, the activity measured using the Bath Ankylosing Spondylitis Disease Activity Index (BASDAI), time receiving anti-TNF therapy, and use of other immunosuppressive therapies.

LLS was defined in patients who showed all the following conditions: 1. A temporal relationship between clinical signs/symptoms and anti-TNFα treatment; 2. At least, one serological test positive for SLE (according to ACR), antinuclear antibodies (ANA), anti-dsDNA, or anti-cardiolipin (ACA); and 3. At least one ACR criteria: arthritis, serositis, hematological disorder, malar erythema.

Moderate SLL was defined in patients who had arthralgia and/or mialgia that subsided with the use of analgesic or nonsteroidal antiinflammatory drugs (NSAIDs) and did not cause the suspension of anti-TNFα therapy.

Severe SSL was defined in patients with arthritis and/or mialgia that did not yield with the use of NSAIDs, as well as in patients that during the course of biological therapy showed: polyserositis, glomerulonephritis, leukopenia, thrombocytopenia, thrombotic disease, hypocomplementemia, fever of unknown origin (FUO) neuropsychtatric SLE.

Measurement of ANA was performed by indirect immunofluorescence (IFI) using HEp-2 cells, and was considered positive when titers of ANA exceeded 1:160 (as measured by HEp-2), and for measurement of anti-dsDNA *Crithidia luciliae *was used.

Patients with ankylosing spondylitis, psoriatic arthritis, enteropathic arthropathy, undifferentiated spondyloarthritis and reactive arthritis (excluded patients with uveitis associated with HLAB27 and juvenile idiopathic arthritis associated with enthesitis) who had received anti-TNFα therapy (infliximab, adalimumab or etanercept) for over 1 year were analyzed for the development of LLS, in addition to measuring the values of ANA ≥ 1:160 (measured by HEp-2) and anti-dsDNA (measured by IIF).

### Statistical analysis

Statistical parameters were described by appropriate central trend and dispersion measures. Values of ANA ≥ 1:160 versus < 1:160 and anti-dsDNA present versus absent, were dichotomized. A descriptive analysis for sex, age, and type of disease was made using mean and range. A multivariate logistic regression analysis was used to assess binary variables: treatment with infliximab, another immunosuppressant treatment. The statistical significance of differences in frequency of variable "signs and symptoms of LLS" was determined by Fisher's exact test and p values were considered significant when < 0.05 (Svejgaard and Ryder, 1994).

## Results

**Figure 1:**
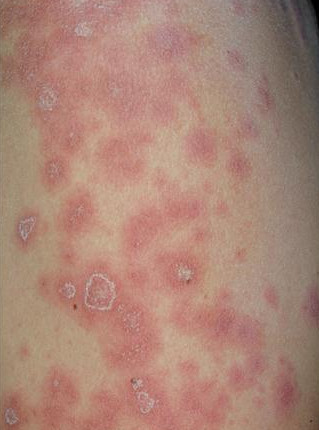
**Papulosquamous lesions on the arm of a patient typical of subacute cutaneous lupus**.

**Figure 2:**
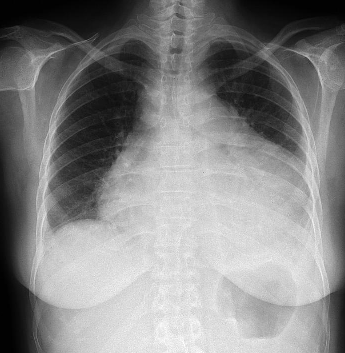
**Pericardial effusion with pleural effusion**.

There was not a high prevalence of LLS in patients with spondyloarthritis treated with anti TNFα therapy. In total, 7.01% of patients treated anti TNF had titers of ANA ≥ 1:160, whereas 3.5% of patients had serum levels of dsDNA. However, only one patient (1.75% n = 1) experienced clinical symptoms of LLS; this was a female patient with a history of psoriatic arthritis of 5 years of evolution, diagnosed with severe psoriasis since the age of 23 years. Besides that the patient was 50 years old and had received anti TNF therapy for over 2 years, she presented annular polycyclic lesions (Figure 1) on the anterior aspect of the arm (subacute cutaneous lupus) with polyserositis (Figure 2), which improved with anti TNF therapy discontinuation and low potency corticosteroid. This patient had ANA > 1:320, presence of anti-dsDNA by IIF and high titers by ELISA (≥ 25 IU/L).

The rest of the patients presenting ANA (+) and anti-dsDNA (+) did not develop SLL, two of them were diagnosed with enteropathic arthritis and one patient with psoriatic arthritis. There was no positivity for ANA in patients with ankylosing spondylitis, reactive arthritis or undifferentiated spondyloarthritis.

## Discussion

The presence of LLS secondary to anti-TNFα therapy in patients with SpA appears to be less frequently observed compared with patients with RA, probably because in the pathogenesis of SpA large numbers of circulating autoantibodies have not been found, may be due to the fact that LLS is an entity associated purely to innate immunity with little central role of B and T cells. In this study signs and symptoms associated with LLS were observed only in one patient with psoriatic arthritis, with severe and long-standing disease, emphasizing that according to medical literature in psoriatic arthritis and psoriasis anti-CCP antibodies have been detected only in 5-12.5% of the population [25].

As for the production of autoantibodies, a fourth hypothesis is now considered as the most solid and has to do with the system of clearance and presentation of apoptotic material by the adhesion molecule CD44. This molecule is involved in phagocytosis of apoptotic neutrophils; TNFα overregulates the cell expression of CD44, which, at start of the biologic therapy with anti-TNFα, a reduction in clearance occurs; [26,27] however, LLS is still a rare disease and there are reviews where risk factors for its development such as ANA presence before treatment, appearance of anti-nucleosome antibody, the antibody isotype and, of course, the genetic polymorphism of anti-TNFα, are considered [28-30].

## Disclosure

E. Puertas-Abreu, None; E. Rodríguez Polanco, None; M. Azocar, None; L.A. Mundarain, None; C. Nuñez-Sotelo, None; R. Montaño, None; F. Herrera Vivas, None; Z. Tovar Noguera, None; F. Marín, None; O. Bellorin, None; J. Rivas, None; E. Toro, None; E. Benzaquen, None; M. Rauseo, None; LA Gutiérrez González, None.

## Authors' contributions

Participated in the recruitment: EP;ER;MA;LM;CN;RM;ZT;FM;OB, JR;ET. Participated in the statistical analysis: LAG;FH. Participated sampling:EB;LAG. All authors read and approved the final manuscript.
